# Supplementary material for: Dynamic Functional Network Connectivity Changes Associated with fMRI Neurofeedback of Right Premotor Cortex
Source: Brain Sci. 2021 Apr 30;11(5):582. doi: 10.3390/brainsci11050582 (PMC8147082; doi:10.3390/brainsci11050582)
Supplement: Supplementary file 1 [file brainsci-11-00582-s001.zip › brainsci-1189603-supplementary.pdf]

Supplementary Materials

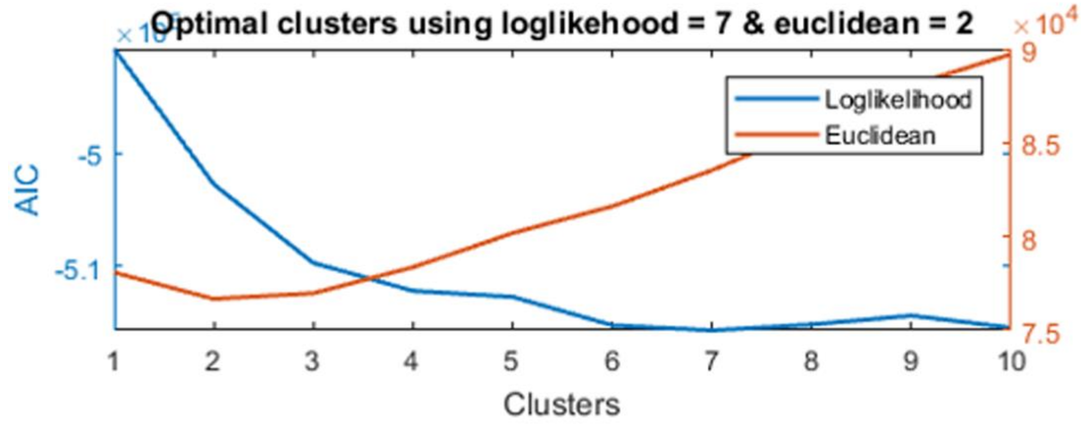

Figure 1. Estimation of optimal clusters by AIC.

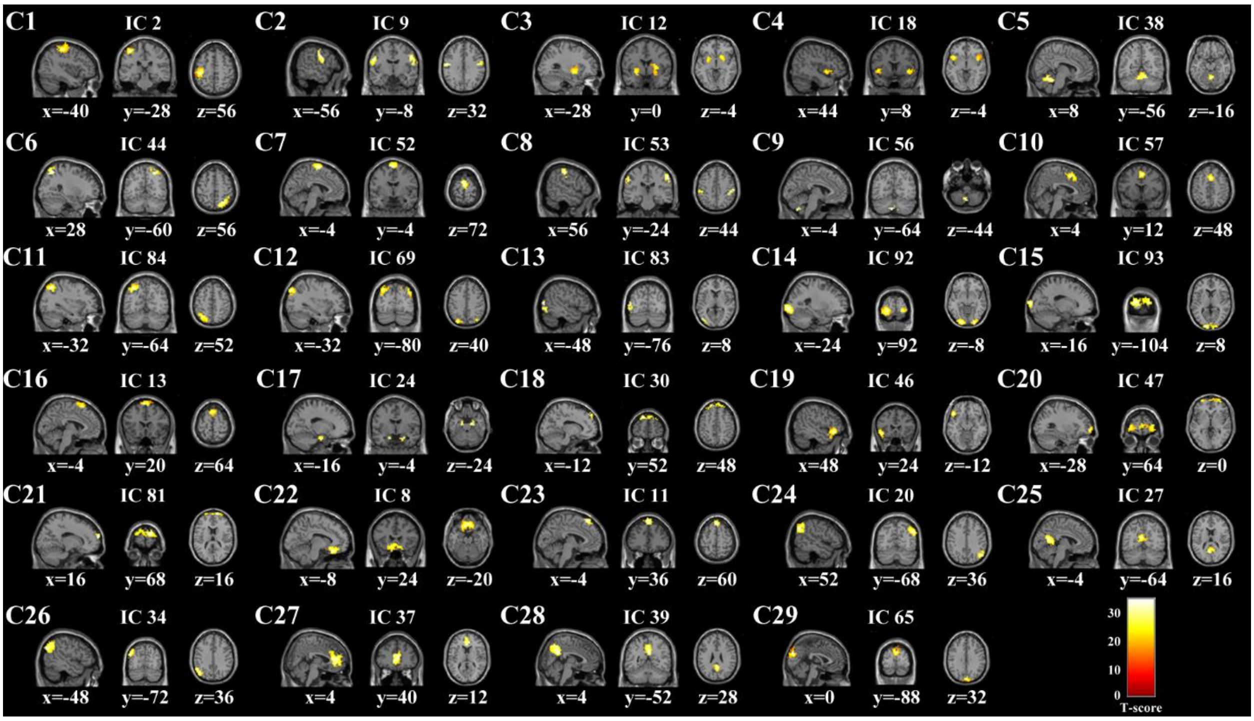

Figure 2. The spatial activation maps of the 29 task-related components that belonged to MN, VN, CCN and DMN.
